# Supplementary material for: Inhibitory Activity of Flavonoids, Chrysoeriol and Luteolin-7-O-Glucopyranoside, on Soluble Epoxide Hydrolase from Capsicum chinense
Source: Biomolecules. 2020 Jan 24;10(2):180. doi: 10.3390/biom10020180 (PMC7072517; doi:10.3390/biom10020180)
Supplement: Supplementary file 1 [file biomolecules-10-00180-s001.pdf]

Inhibitory activity of flavonoids, chrysoeriol and luteolin-7-*O*-glucopyranoside, on soluble epoxide hydrolase

Jang Hoon Kim and Chang Hyun Jin*

Advanced Radiation Technology Institute, Korea Atomic Energy Research Institute, Jeongeup, Jeollabuk-do 56212, Republic of Korea

* Corresponding author:

Dr. Chang Hyun Jin

Tel., +82-63-570-3162; Fax, +82-63-570-3159 ; E-mail address: chjin@Kaeri.re.kr

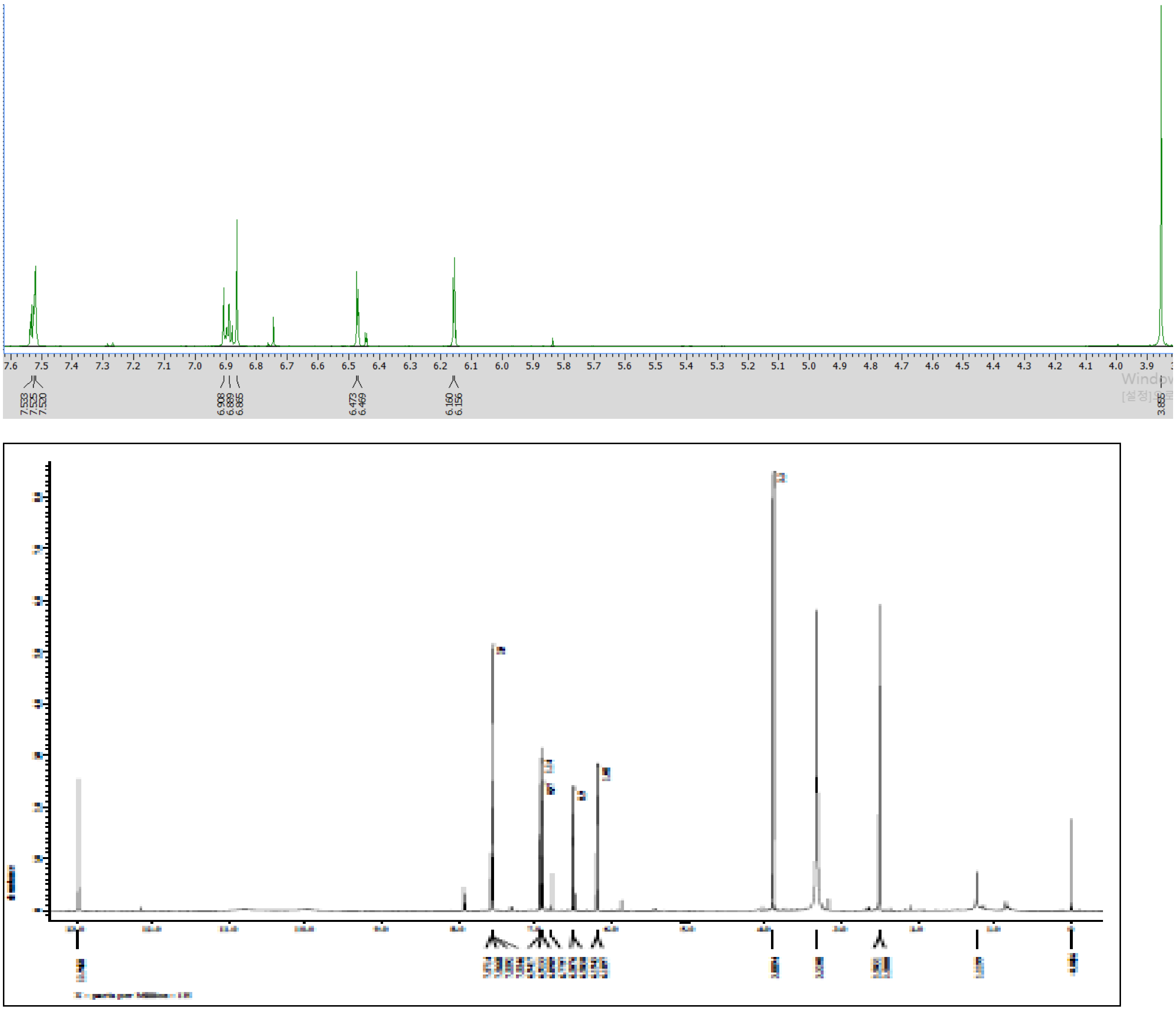

Figure S1. ^{1}H-NMR of compound **1**.

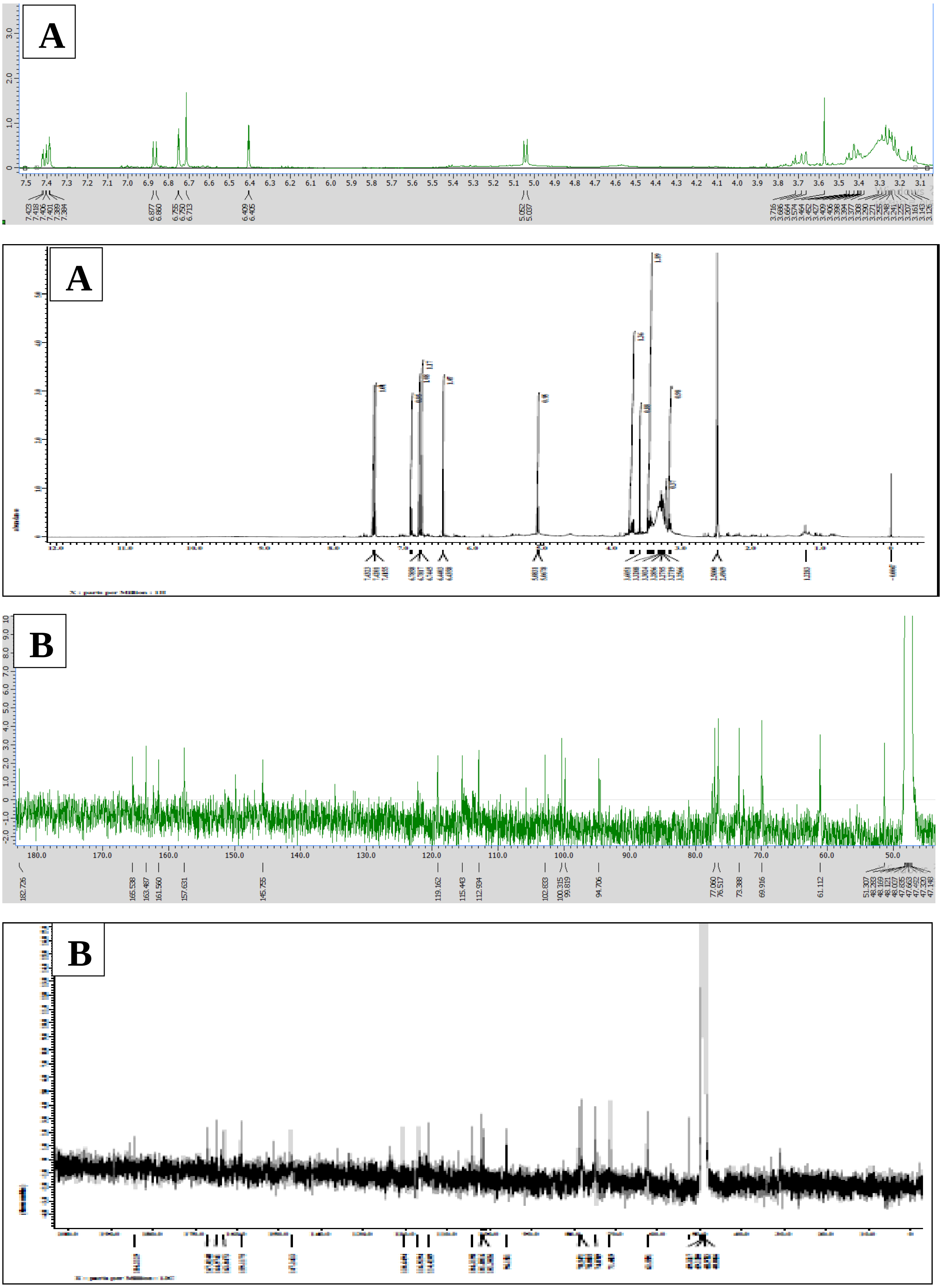

Figure S2. ^{1}H(A) and^{13}C(B)-NMR of compound **2**.

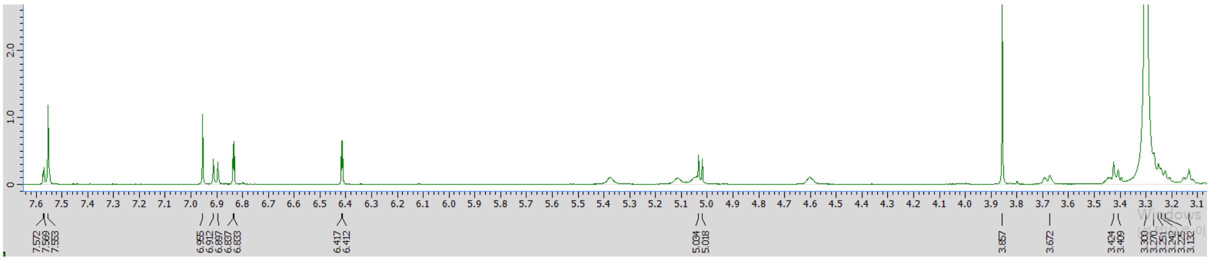

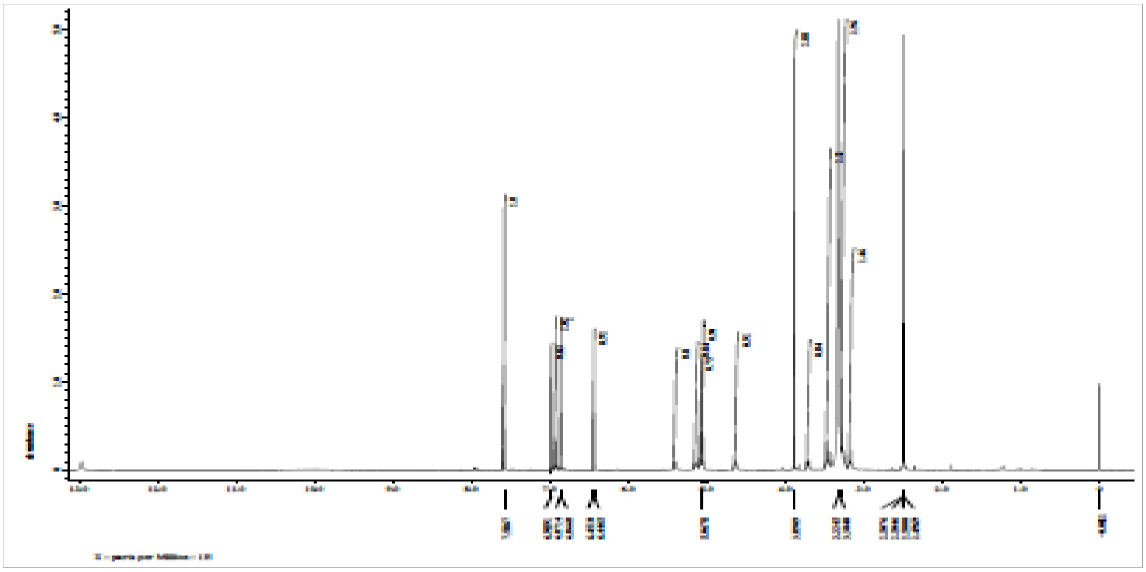

Figure S3.^{13}C-NMR of compound **3**.
